# Distal Colon Motor Coordination: The Role of the Coloanal Reflex and the Rectoanal Inhibitory Reflex in Sampling, Flatulence, and Defecation

**DOI:** 10.3389/fmed.2021.720558

**Published:** 2021-09-06

**Authors:** Jan D. Huizinga, Lijun Liu, Ashley Barbier, Ji-Hong Chen

**Affiliations:** Department of Medicine, Division of Gastroenterology, Farncombe Family Digestive Health Research Institute, McMaster University, Hamilton, ON, Canada

**Keywords:** colonic motility, colonic motility and disorders, RAIR, coloanal reflex, anal sphincter

## Introduction

Chronic constipation can be associated with a colonic motility disorder and/or obstructive defecation. An obstructive defecation disorder is suspected if the patient uses digital evacuation, performs excessive straining, and/or has a sensation of incomplete evacuation. A study using radiopaque shapes can reveal abnormal transit; colonic manometry can inform on abnormal colonic motor patterns or aberrant autonomic reflexes; anorectal manometry can reveal dyssynergia. Here, we discuss the physiological and clinical aspects of anal sphincter relaxation associated with continence, flatulence, and defecation, focussing on the rectoanal inhibitory reflex (RAIR) and the coloanal reflex.

The impetus for this opinion paper was to discuss the conclusion by Pucciani and Trafeli (1) that parameters of a *normal* RAIR, can reveal the *pathophysiology* of obstructive defecation. Their methodology involved a detailed evaluation of the parameters of the RAIR, in a cohort of 58 patients. The patients reported a normal bowel movement frequency and stool form was normal, but most performed digital evacuation with excessive straining. The patients as a group had normal minimum and maximum anal sphincter pressures, the maximum tolerated volume was normal and so was the threshold for initiation of the RAIR. The authors concluded that the RAIR was impaired in these patients because the average internal anal sphincter (IAS) relaxation was 74% upon rectal distention by a 60 ml balloon compared to 92% in their control group of 20 subjects. Although the difference was statistically significant, it is not clinically relevant since 74% relaxation associated with the RAIR is normal, if not excellent, according to international standards (2, 3). In addition, the relaxation evoked by the RAIR was deemed too short because of excessive external anal sphincter (EAS) contraction *prior* to the relaxation (what was named a rectoanal excitatory reflex RAER) (1). The relevance of this short transient excitatory reflex that gave on average a 13 mmHg pressure increase is unclear because it does not prevent relaxation, and it is therefore fundamentally different from the type of dyssynergia where anal sphincter contraction *prevents* proper relaxation (4); this RAER is indeed considered normal (5, 6).

The relaxation that allows defecation and gas expulsion involves a proper sensation to initiate reflex relaxation, a sufficient relaxation of the IAS and the EAS, and the absence of counteracting contraction of the pelvic floor muscles and anal sphincters. It is the coloanal reflex that can produce full relaxation of both the IAS and the EAS, whereas the RAIR relaxes the IAS to allow sampling of content in the proximal anal canal.

## The Value of Assessing the RAIR

The RAIR test is executed to assess the response of the IAS to rapid and transient graded distentions of the rectum induced by a balloon. It is the classic test to discover Hirschsprung's disease: the inability of IAS relaxation in a newborn who does not pass stool suggests abnormal intrinsic inhibitory innervation of the anal canal, likely as the result of distal colon aganglionosis. In addition, nitrergic innervation of the IAS can be absent in children with Hirschsprung's disease (7). The RAIR can also be absent in asymptomatic patients with dys-ganglionosis, post-circular myotomy, and lower anterior resections (8).

The RAIR, in response to balloon distention is usually interpreted as absent or present, and a relaxation of 20% or more of baseline pressure is considered normal. In a characterization of the RAIR in healthy subjects by Rao and co-workers, a transient distention by a 70 ml balloon at a peak anal sphincter pressure of 70–80 mmHg for 30 s, caused a maximal relaxation of 47 mmHg, leaving a minimum pressure of 32 mmHg, proposed to be maintained by the EAS and puborectalis (4).

Anal sphincter relaxation in response to the RAIR is primarily due to activation of enteric nitrergic nerves (9, 10) and hence restricted to the IAS. In a study by Beuret-Blanquart et al. (11), all patients with complete transection of the spinal cord exhibited the RAIR. In patients with absence of sympathetic control (transection between T9 and L2), the RAIR was completely normal. In patients with parasympathetic lesions (transection between S2 and S4), the RAIR was present, but amplitude and duration were not correlated with distention volume suggesting a regulatory role by the parasympathetic nucleus (11). Similarly, in patients with sacral agenesis, the RAIR is present but not modulated by different levels of rectal distention (12). Hence, the rapid transient distention of the rectum when a RAIR is assessed, will stimulate both enteric and spinal sensory nerves and the subsequent relaxation is mediated by enteric inhibitory nerves with or without involvement of parasympathetic nerves acting on the enteric inhibitory nerves (13).

The RAIR is sometimes called a defecation reflex, but it is more aptly called a sampling reflex; relaxation of the proximal IAS allows filling of the proximal anal canal which gives the central nervous system a chance to stop, or go ahead, with flatulence or defecation, hence it is involved in maintaining continence. The RAIR does not relax the EAS for expulsion. It is illustrative that patients with Hirschsprung's disease, after successful surgery, still do not have a RAIR but have normal defecation, indicating that the RAIR is not needed for defecation (14, 15).

## Relaxation and Dyssynergia

Dyssynergia involves the inability to relax the anal sphincters because of an autonomic dysfunction to prevent relaxation and/or the presence of counteracting contractions of the anal sphincters and/or pelvic floor muscles; hence reflex relaxation is prevented that may indicate abnormal sensory or motor innervation, or may indicate an acquired behavioral problem that can be rectified with biofeedback (16, 17). Dyssynergia is identified when *several* tests provide evidence, since the reflexes evoked in the laboratory are often abnormal, even in healthy subjects, because of the unnatural testing conditions. With anorectal manometry, relaxation can be observed in response to rapid transient balloon distention. Relaxation can also be evaluated by asking the patient to bear down, simulating defecation during anorectal manometry or digital examination. With dyssynergia, tightening of the sphincters may occur, increasing pressure. A water filled balloon inserted in the rectum is normally expelled within a minute but with dyssynergia it may be delayed or absent. It should be noted that the patient will strain in response to bearing down or to expel the balloon, hence, these are tests of straining-assisted defecation, not necessarily a test of a normal defecation reflex. Of note, chronic excessive straining maybe a *cause* of dyssynergia (2). Dyssynergia can also be observed with defecography although it is mostly qualitative, with fluoroscopic defecography preferred over MRI defecography, since the fluoroscopic assessment is done in a more physiological condition where patients can maneuver according to their habits (18).

## The Coloanal Reflex

**Figure 1:**
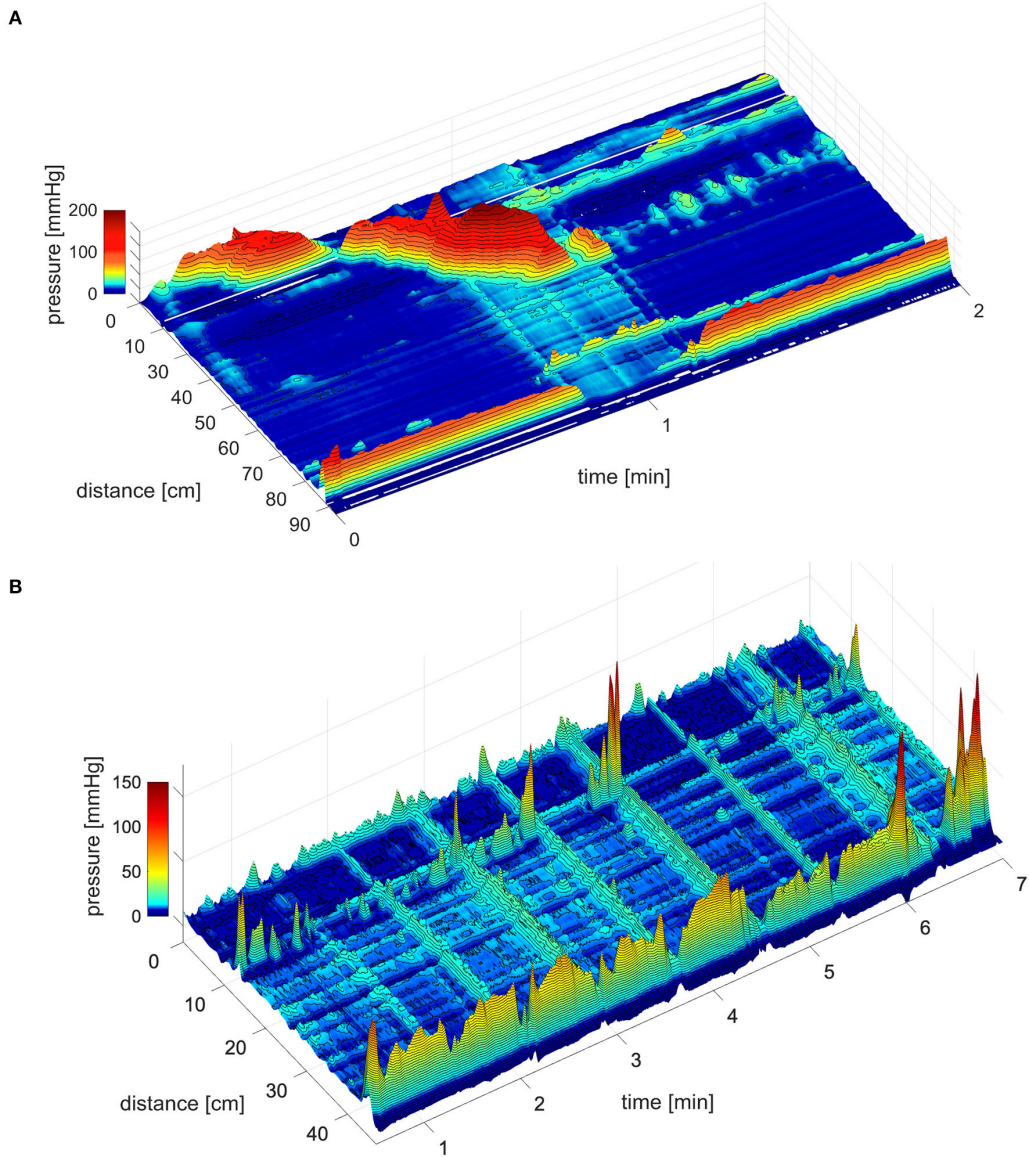
The coloanal reflex. **(A)** The coloanal reflex associated with an HAPW followed by a simultaneous pressure wave that enters the rectum followed by complete relaxation of the anal sphincters. 0 cm is in the proximal colon, the anal sphincter is at 85 cm. The white line represents a 10 cm section where a balloon is situated. From Milkova et al. (24). **(B)** Simultaneous pressure waves followed by anal sphincter relaxation, in this particular session, the strength of the relaxation was related to the amplitude of the SPW. However, even low amplitude SPWs can generate complete AS relaxation. From Chen et al. (25).

The coloanal reflex refers to the relaxation of the anal sphincters in response to a propulsive motor pattern of the colon (6, 14, 19–23). The relaxation can be evoked by a high-amplitude propagating pressure wave (HAPW) generated in the proximal colon, that does not visibly come anywhere near the rectum (24). It can be evoked when a simultaneous pressure wave (SPW) enters the rectum associated with gas expulsion, evoking pressures during the SPW in the colon that rarely exceed 20 mmHg (21, 25), or it can be evoked when an HAPW travels through the transverse or descending colon and changes into an SPW that instantly relaxes the sphincters. The coloanal reflex is an essential part of defecation and flatulence. The coloanal reflex can relax both sphincters completely (24, 25) (Figure 1) in contrast to the RAIR that involves relaxation of the proximal IAS. The HAPW takes part in the preparatory phase of defecation (26) and can be part of the act of defecation itself (27). High-amplitude propagating pressure waves and SPWs are generated as part of reflexes such as the gastrocolic reflex and autonomic reflexes mediated by vagal and sacral parasympathetic nerves in response to colonic or rectal distention (24, 25, 28–30).

The IAS relaxation as a consequence of the coloanal reflex (Figure 1), is mediated by intrinsic activation of enteric nitrergic nerves, parasympathetic activation of nitrergic nerves, and nitric oxide release from nNOS+ parasympathetic nerve fibers (29). Internal anal sphincter tone is primarily myogenic, a summation of phasic contractions of the musculature orchestrated by intramuscular interstitial cells of Cajal (31). Internal anal sphincter pressure is normal in patients with isolated sacral agenesis (12). Anal sphincter relaxation in response to a proximal HAPW is often not accompanied by pressure generation in the descending colon and rectum (22, 24), hence, it involves a spinal reflex. The varicose sympathetic innervation of the IAS musculature contributes to tone and high spinal nerve block reduces IAS pressure (32, 33). Hence, it is conceivable that overexcitation of sympathetic nerves may inhibit normal relaxation and so contribute to dyssynergia.

The relaxation of the EAS as a consequence of the coloanal reflex (Figure 1), is likely facilitated by autonomous inhibition of motor neurons in Onuf's nucleus (25). It is an autonomous reflex associated with autonomic control of EAS tone (34). The EAS tone is achieved by the (unusual) tonic activity of a somatic nerve, the pudendal nerve. External anal sphincter tone was not normal in patients with isolated sacral agenesis and these patients were incontinent (12). Relaxation of the EAS occurs autonomously when a propulsive motor pattern enters the rectum as part of the coloanal reflex (35) (Figure 1) just as autonomic contraction of the EAS is an important part of maintaining continence (34).

Assessment of the coloanal reflex with high-resolution colonic manometry, can identify distal colon discoordination and dyssynergia, when the anal sphincters fail to relax in association with propulsive motor patterns or when excessive contraction of the anal sphincters and/or the sphincter of O'Beirne is observed (36). The sphincter of O'Beirne is the specialized musculature at the rectosigmoid junction that can play a critical role in constipation (19, 36). The voluntary contraction of the EAS can be evoked as a reaction to an urge to defecate following the generation of an HAPW or the arrival of a SPW into the rectum, to maintain continence. However, excessive, aberrant autonomous contraction of anal sphincters, pelvic floor muscles, and/or the sphincter of O'Beirne that prevent reflex relaxation, constitutes autonomous dyssynergia (36). The RAIR, on the other hand, is a response to a c*onscious* experience of rectal distention that can also reveal dyssynergia. Low or high-resolution anorectal manometry can assess various aspects of rectoanal function such as the degree of IAS pressure inhibition, the slope of inhibition and recovery, any excitatory responses, etc. (37). High-resolution manometry, in addition, can inform about detailed pressure differences throughout the anal canal. These details may inform about the physiological parameters of the reflex and may reveal differences between constipation and incontinence (37), but lack of standardization and the likelihood of a large variability in control values, makes clinical relevance uncertain.

## Discussion

The RAIR in daily life is a sampling reflex that allows filling of the proximal anal canal to allow us to make a decision whether or not to accept expulsion of gas or stool; it is an integral part of our ability to maintain continence and can be the first step toward defecation or flatulence. The manometric parameters of a normal RAIR do not reveal the pathogenesis of obstructed defecation. The coloanal reflex is critical for complete defecation or gas expulsion as it involves relaxation of both the IAS and EAS, and absence will lead to distal colon dysmotility. An abnormal coloanal reflex is an autonomic nervous system dysfunction that can be the primary pathophysiology in outlet obstruction.

## Author Contributions

This review was conceived and written by JH and J-HC. LL and AB made substantial contributions. All authors approved the final version.

## Conflict of Interest

The authors declare that the research was conducted in the absence of any commercial or financial relationships that could be construed as a potential conflict of interest.

## Publisher's Note

All claims expressed in this article are solely those of the authors and do not necessarily represent those of their affiliated organizations, or those of the publisher, the editors and the reviewers. Any product that may be evaluated in this article, or claim that may be made by its manufacturer, is not guaranteed or endorsed by the publisher.

## Abbreviations

RAIR: rectoanal inhibitory reflex
HAPW: high amplitude propagating pressure wave
SPW: simultaneous pressure wave
EAS: external anal sphincter
IAS: internal anal sphincter.

## References

[1] Pucciani F Trafeli M. Sampling reflex: pathogenic role in functional defecation disorder. Tech Coloproctol. (2021) 25:521–30. 10.1007/s10151-020-02393-5 33587211

[2] Rao SS Patcharatrakul T. Diagnosis and treatment of dyssynergic defecation. J Neurogastroenterol Motil. (2016) 22:423–35. 10.5056/jnm16060 27270989 PMC4930297

[3] Lee TH Bharucha AE. How to perform and interpret a high-resolution anorectal manometry test. J Neurogastroenterol Motil. (2016) 22:46–59. 10.5056/jnm15168 26717931 PMC4699721

[4] Cheeney G Nguyen M Valestin J Rao SS. Topographic and manometric characterization of the recto-anal inhibitory reflex. Neurogastroenterol Motil. (2012) 24:e147–54. 10.1111/j.1365-2982.2011.01857.x 22235880 PMC4566956

[5] Sangwan YP Coller JA Barrett RC Murray JJ Roberts PL Schoetz DJ. Distal rectoanal excitatory reflex: a reliable index of pudendal neuropathy? Dis Colon Rectum. (1995) 38:916–20. 10.1007/BF02049725 7656737

[6] Crowell MD. Pathogenesis of slow transit and pelvic floor dysfunction: from bench to bedside. Rev Gastroenterol Disord. (2004) 4(Suppl 2):S17–27. 15184815

[7] Kobayashi H Hirakawa H Puri P. Abnormal internal anal sphincter innervation in patients with Hirschsprung's disease and allied disorders. J Pediatr Surg. (1996) 31:794–9. 10.1016/S0022-3468(96)90136-0 8783106

[8] Carrington EV Heinrich H Knowles CH Fox M Rao S Altomare DF. The international anorectal physiology working group (IAPWG) recommendations: Standardized testing protocol and the London classification for disorders of anorectal function. Neurogastroenterol Motil. (2020) 32:e13679. 10.1111/nmo.13679 31407463 PMC6923590

[9] De Lorijn F De Jonge WJ Wedel T Vanderwinden JM Benninga MA Boeckxstaens GE. Interstitial cells of Cajal are involved in the afferent limb of the rectoanal inhibitory reflex. Gut. (2005) 54:1107–13. 10.1136/gut.2004.051045 16009682 PMC1774907

[10] Bharucha AE. Pelvic floor: anatomy and function. Neurogastroenterol. Motil. (2006) 18:507–19. 10.1111/j.1365-2982.2006.00803.x 16771766

[11] Beuret-Blanquart F Weber J Gouverneur JP Demangeon S Denis P. Colonic transit time and anorectal manometric anomalies in 19 patients with complete transection of the spinal cord. J Auton Nerv Syst. (1990) 30:199–207. 10.1016/0165-1838(90)90251-D 2229888

[12] Morera C Nurko S. Rectal manometry in patients with isolated sacral agenesis. J Pediatr Gastroenterol Nutr. (2003) 37:47–52. 10.1097/00005176-200307000-00008 12827005

[13] Buntzen S Nordgren S Hultén L Delbro D. The role of nitric oxide in the acetylcholine-induced relaxation of the feline internal anal sphincter, *in vitro*. Scand J Gastroenterol. (1996) 31:1189–94. 10.3109/00365529609036909 8976011

[14] Sintusek P Rybak A Mutalib M Thapar N Borrelli O Lindley KJ. Preservation of the colo-anal reflex in colonic transection and post-operative Hirschsprung's disease: Potential extrinsic neural pathway *Neurogastroenterol*. Motil. (2018) 31:e13472. 10.1111/nmo.13472 30288858

[15] Demirbag S Tiryaki T Purtuloglu T. Importance of anorectal manometry after definitive surgery for Hirschsprung's disease in children. Afr J Paediatr Surg. (2013) 10:1–4. 10.4103/0189-6725.109370 23519848

[16] Rao SS Mudipalli RS Stessman M Zimmerman B. Investigation of the utility of colorectal function tests and Rome II criteria in dyssynergic defecation (Anismus). Neurogastroenterol Motil. (2004) 16:589–96. 10.1111/j.1365-2982.2004.00526.x 15500515

[17] Rao SS Benninga MA Bharucha AE Chiarioni G Di Lorenzo C Whitehead WE. ANMS-ESNM position paper and consensus guidelines on biofeedback therapy for anorectal disorders. Neurogastroenterol Motil. (2015) 27:594–609. 10.1111/nmo.12520 25828100 PMC4409469

[18] Palmer SL Lalwani N Bahrami S Scholz F. Dynamic fluoroscopic defecography: updates on rationale, technique, and interpretation from the Society of Abdominal Radiology Pelvic Floor Disease Focus Panel. Abdom Radiol (NY). (2021) 46:1312–22. 10.1007/s00261-019-02169-y 31375862

[19] Chen J-H Nirmalathasan S Pervez M Milkova N Huizinga JD. The sphincter of O'Beirne - part 1: study of 18 normal subjects. Dig Dis Sci. (2021). 10.1007/s10620-020-06657-w. [Epub ahead of print]. 33462748

[20] Malcolm A Camilleri M. Coloanal motor coordination in association with high-amplitude colonic contractions after pharmacological stimulation. Am J Gastroenterol. (2000) 95:715–9. 10.1111/j.1572-0241.2000.01840.x 10710063

[21] Corsetti M Pagliaro G Demedts I Deloose E Gevers A Scheerens C. Pan-colonic pressurizations associated with relaxation of the anal sphincter in health and disease: a new colonic motor pattern identified using high-resolution manometry. Am J Gastroenterol. (2017) 112:479–89. 10.1038/ajg.2016.341 27596695

[22] Rodriguez L Siddiqui A Nurko S. Internal anal sphincter relaxation associated with bisacodyl-induced colonic high amplitude propagating contractions in children with constipation: a colo-anal reflex? Neurogastroenterol Motil. (2012). 24:1023–e545. 10.1111/j.1365-2982.2012.01965.x 22757618 PMC3465462

[23] Reboa G Arnulfo G Frascio M Di Somma C Pitto G Berti-Riboli E. Colon motility and colo-anal reflexes in chronic idiopathic constipation. Effects of a novel enterokinetic agent cisapride. Eur J Clin Pharmacol. (1984) 26:745–8. 10.1007/BF00541936 6489414

[24] Milkova N Parsons SP Ratcliffe E Huizinga JD Chen JH. On the nature of high-amplitude propagating pressure waves in the human colon. Am J Physiol Gastrointest Liver Physiol. (2020) 318:G646–60. 10.1152/ajpgi.00386.2019 32068445 PMC7191456

[25] Chen J-H Parsons SP Shokrollahi M Wan A Vincent AD Yuan Y. Characterization of simultaneous pressure waves as biomarkers for colonic motility assessed by high-resolution colonic manometry. Front Physiol. (2018) 9:1248. 10.3389/fphys.2018.01248 30294277 PMC6159752

[26] Bampton PA Dinning PG Kennedy ML Lubowski DZ deCarle D Cook IJ. Spatial and temporal organization of pressure patterns throughout the unprepared colon during spontaneous defecation. Am J Gastroenterol. (2000) 95:1027–35. 10.1111/j.1572-0241.2000.01839.x 10763955

[27] Rao SS Sadeghi P Beaty J Kavlock R Ackerson K. Ambulatory 24-h colonic manometry in healthy humans. Am J Physiol Gastrointest Liver Physiol. (2001) 280:G629–39. 10.1152/ajpgi.2001.280.4.G629 11254489

[28] De Groat WC Krier J. The sacral parasympathetic reflex pathway regulating colonic motility and defaecation in the cat. J Physiol. (1978) 276:481–500. 10.1113/jphysiol.1978.sp012248 650474 PMC1282439

[29] Browning KN Travagli RA. Central control of gastrointestinal motility. Curr Opin Endocrinol Diabetes Obes. (2019) 26:11–6. 10.1097/MED.0000000000000449 30418187 PMC6512320

[30] Smith-Edwards KM Najjar SA Edwards BS Howard MJ Albers KM Davis BM. Extrinsic primary afferent neurons link visceral pain to colon motility through a spinal reflex in mice. Gastroenterology. (2019). 157:522.e2–36.e2. 10.1053/j.gastro.2019.04.034 31075226 PMC6995031

[31] Keef K Cobine C. Generation of spontaneous tone by gastrointestinal sphincters. Adv Exp Med Biol. (2019) 1124:47–74. 10.1007/978-981-13-5895-1_2 31183822

[32] Carlstedt A Nordgren S Fasth S Appelgren L Hulten L. Sympathetic nervous influence on the internal anal sphincter and rectum in man. Int J Colorectal Dis. (1988) 3:90–5. 10.1007/BF01645312 3411187

[33] Cobine C. The Roles of Interstitial Cells in the Regulation of Motility in the Rectoanal Region. Dissertation, University of Nevada (2011).

[34] Broens PM Penninckx FM Ochoa JB. Fecal continence revisited: the anal external sphincter continence reflex. Dis Colon Rectum. (2013) 56:1273–81. 10.1097/DCR.0b013e3182a42d16 24105003

[35] Tan W Lee G Chen J-H Huizinga JD. Relationships between distention-, butyrate- and pellet-induced stimulation of peristalsis in the mouse colon. Front Physiol. (2020) 11:109. 10.3389/fphys.2020.00109 32132933 PMC7040375

[36] Chen J-H Collins SM Milkova N Pervez M Nirmalathasan S Tan W. The Sphincter of O'Beirne-part 2: report of a case of chronic constipation with autonomous dyssynergia. Dig Dis Sci. (2021). 10.1007/s10620-020-06723-3. [Epub ahead of print]. 33462747

[37] Zbar AP Aslam M Gold DM Gatzen C Gosling A Kmiot WA. Parameters of the rectoanal inhibitory reflex in patients with idiopathic fecal incontinence and chronic constipation. Dis Colon Rectum. (1998) 41:200–8. 10.1007/BF02238249 9556245

